# Toll-Like Receptor 4 Stimulation before or after *Streptococcus pneumonia*e Induced Sepsis Improves Survival and Is Dependent on T-Cells

**DOI:** 10.1371/journal.pone.0086015

**Published:** 2014-01-21

**Authors:** Edgar Musie, Christopher C. Moore, Edward N. Martin, W. Michael Scheld

**Affiliations:** 1 University of Venda, Department of Microbiology, Venda, South Africa; 2 University of Virginia, Department of Medicine, Division of Infectious Diseases and International Health, Charlottesville, Virginia, United States of America; University of Cincinnati, United States of America

## Abstract

**Introduction:**

Endotoxin tolerance improves outcomes from gram negative sepsis but the underlying mechanism is not known. We determined if endotoxin tolerance before or after pneumococcal sepsis improved survival and the role of lymphocytes in this protection.

**Methods:**

Mice received lipopolysaccharide (LPS) or vehicle before or after a lethal dose of *Streptococcus pneumonia*e. Survival, quantitative bacteriology, liver function, and cytokine concentrations were measured. We confirmed the necessity of Toll-like receptor 4 (TLR4) for endotoxin tolerance using C3H/HeN (TLR4 replete) and C3H/HeJ (TLR4 deficient) mice. The role of complement was investigated through A/J mice deficient in C5 complement. CBA/CaHN-Btk^xid/^/J mice with dysfunctional B cells and Rag-1 knockout (KO) mice deficient in T and B cells delineated the role of lymphocytes.

**Results:**

Endotoxin tolerance improved survival from pneumococcal sepsis in mice with TLR4 that received LPS pretreatment or posttreatment. Survival was associated with reduced bacterial burden and serum cytokine concentrations. Death was associated with abnormal liver function and blood glucose concentrations. Endotoxin tolerance improved survival in A/J and CBA/CaHN-Btk^xid/^/J mice but not Rag-1 KO mice.

**Conclusions:**

TLR4 stimulation before or after *S. pneumoniae* infection improved survival and was dependent on T-cells but did not require an intact complement cascade or functional B cells.

## Introduction

Sepsis remains an important cause of global deaths and is associated with a dysregulated immune response leading to organ failure and death [1]. Many of these cases are due to pneumococcal infection including community acquired pneumonia and meningitis [2]. Although antibiotic resistance occurs in *Streptococcus pneumoniae*, most infections should be adequately treated by available antibiotics [3]. Despite this, patients who develop severe sepsis or septic shock from pneumococcal infection still have relatively poor outcomes [4]. These poor outcomes may be related to the host response to infection.

The innate immune system allows a rapid response to infection via toll-like receptors (TLRs) and other pathogen recognition receptors which trigger down-stream inflammation. Cell-wall components (peptidoglycan and lipoteichoic acid) of the pneumococcus are recognized by TLR2 and the inflammatory response of murine macrophages to the pneumococcal toxin pneumolysin depends on TLR4 [5]. Additionally, mice that lack TLR4 are significantly more susceptible to invasive disease and death following infection with pneumococci, compared to wild-type mice [6], [7]. These data suggest an important protective role for both TLR2 and TLR4 in the host response to pneumococcal infection.

Although the innate immune response is important to contain infections, dysregulation of the response can lead to deleterious outcomes. It has been previously shown that administration of low concentrations of cell-wall components such as lipopolysaccharide (LPS) or lipoprotein confers immune tolerance resulting in resistance to subsequent normally lethal exposure to cell-wall proteins or live bacteria [8]–[13]. This endotoxin tolerance is associated with an attenuated inflammatory response and has previously been attributed to a down-regulation of macrophage responsiveness [14]–[16].

It is not currently known whether the beneficial effects of endotoxin tolerance extend to pneumococcal infection or which other components of cellular immunity are required. Recent studies have suggested an important role for T and B-cells in the immunopathogenesis of sepsis [17], [18]. Therefore this study was designed to determine if the survival benefit of endotoxin tolerance would extend to a *Streptococcus pneumoniae* infection and whether T and B cells are required for the beneficial effects of endotoxin tolerance.

## Materials and Methods

### Experimental animals and reagents

Female C57BL/6, CBA/CaHN-Btk^xid/^/J, A/J, Rag-1 KO (Jackson laboratories, Bar Harbor), C3H/HeN and C3H/HeJ (Charles River) mice, 8–10 weeks of age and weighing approximately 20 grams each, were kept in a sterile environment prior to the start of experiments. The mice were provided standard chow and water *ad libitum* in a 12-hr light/dark cycle. We used C3HeJ mice which are TLR4 deficient along with C3H/HeN mice which are TLR4 replete to determine whether the survival benefit of LPS pretreatment is dependent upon TLR4. To investigate the involvement of the activity of complement components in endotoxin tolerance and control of bacterial infection we used C5 deficient A/J mice. To evaluate the role of B cells and antibody production in endotoxin tolerance we used CBA/CaHN-Btk^xid/^/J mice which express the X-linked immunodeficiency gene *xid*. This B-lymphocyte-specific defect results in an inability to launch an antibody response to thymus-independent type II antigens. They have low serum IgM and IgG3 and a reduced number of B-cells. Moreover, the B-cells that are present have a reduced surface IgM to IgD ratio, which suggests a disorder in B-cell maturation. We did not perform any random testing to confirm dysfunctionality or functionality in the modified strains prior to including them in our experiments.

Ultrapure Escherichia coli 0111:B4 LPS was purchased from InvivoGen (San Diego, USA). In all cases, vehicle used was phosphate buffered saline. Isothesia-isoflurane was purchased from Butler Schein Animal Health (Dublin, OH). The protocol used in the present study was approved by the Animal Care and Use Committee, University of Virginia, USA (Protocol 1096). Our studies were designed to investigate methods to improve survival from sepsis, so we used the clinical endpoint of death for these studies; however, in order to minimize suffering, if a mouse was found in a moribund state as defined by the University of Virginia Institutional Animal Care and Use Committee they were anesthetized with ketamine/xylazine prior to cervical dislocation. Animals were monitored every 6 hours after the onset of experimental sepsis.

### Bacteria

*Streptococcus pneumoniae* ATCC 6302™ from the American Type Culture Collection (Manassas, VA, USA) were grown as previously described [19]. Briefly, bacterial stock stored at -70°C was thawed and resuscitated by two consecutive passes on 5% sheep blood Tryptic soy agar (TSA) supplemented with 0.5% yeast extract and incubated for 15 hours under 5% CO_2_ atmosphere. Colonies from the second plate were inoculated into 10 ml of supplemented Brain Heart Infusion broth, followed by 3 successive 1∶10 dilutions into identical liquid media. All tubes were incubated for 12 hours under 5% CO_2_ atmosphere. One ml of bacterial suspension taken from the most diluted tube with visible growth was inoculated into 9 ml of supplemented Brain Heart Infusion broth and incubated for 3 hours at 37°C under 5% CO_2_ atmosphere. Optical density was measured to obtain the desired concentration (Vitek 15 = 10^8^ CFU/ml) and serial dilutions were plated on 5% sheep blood TSA to ensure final concentration of approximately 10^8^ CFU/ml.

### Infection and survival

Mice received 5 intravenous (iv) injections of 10 ug of highly purified LPS or vehicle at 12 hour intervals 48 hours prior to iv administration of a lethal dose (2×10^6^ cfu) of *S. pneumoniae* in a 50 µl suspension. In some experiments LPS was given 3 or 6 hours after bacterial challenge and then every 12 hours thereafter for two days. In these experiments, ceftriaxone was also given at 6 hours after bacterial challenge. Controls for all experiments received PBS instead of LPS. Survival was monitored for 5 days after bacterial challenge.

### Quantitative bacteriology

Mice were anaesthetized with Isothesia-isoflurane™ and blood was collected by cardiac puncture prior to sacrifice via cervical dislocation. Lungs were dissected carefully and excised, and then separately homogenized in 25 ml of saline by teasing with a stainless mesh at room temperature. Blood and lung homogenates collected at t = +6 hours were serially diluted 10-fold with sterile physiological saline or PBS, and 0.2 ml samples of the various dilutions were inoculated onto 5% sheep blood TSA. The plates were incubated at 37°C 5% CO_2_ overnight. Colonies were enumerated and bacterial counts were expressed as the log number of CFU per milliliter of blood.

### Biochemical assays for liver function

Serum concentrations of glucose, aspartate aminotransferase (AST), and alanine aminotransferase (ALT) were determined in blood samples collected 6 hours after *S. pneumoniae* inoculation. Heparinized blood samples were spun at 10, 000×*g* for 15 minutes then serum samples were collected and frozen at −70°C until blood chemistry activities were analyzed. Biochemical assays for liver function were analyzed by the Clinical Pathology Laboratory at the University of Virginia Medical Center by use of an automated spectrophotometric assay (Abbott Architect C8000).

### Cytokine measurements

Blood samples from untreated controls and LPS pretreated mice were collected at different time points after bacterial infection. Individual sera were separated from blood by centrifugation and stored at −70°C until cytokine assays were performed. Serum cytokines were determined by using protein-based multiplex immunoassay system (Bio-Rad Laboratories, Hercules, CA) in duplicates per sample according to the manufacturer's instructions.

### Statistical analysis

Culture data from each time point were expressed as mean log CFU ± standard deviation (SD) for each group. Statistical comparisons of cytokine values were done by a 2 tailed Student's t-Test (Microsoft Excel software, Microsoft Corporation, Redmond, WA). Survival data were plotted and statistical analysis for survival studies was performed using the log-rank test with GraphPad Prism, version 5.0 for windows (GraphPad PRISM Software, San Diego, CA). Data are displayed as means ± SD unless otherwise stated. Differences were considered statistically significant when p<0.05.

## Results

### Survival

**Figure 1:**
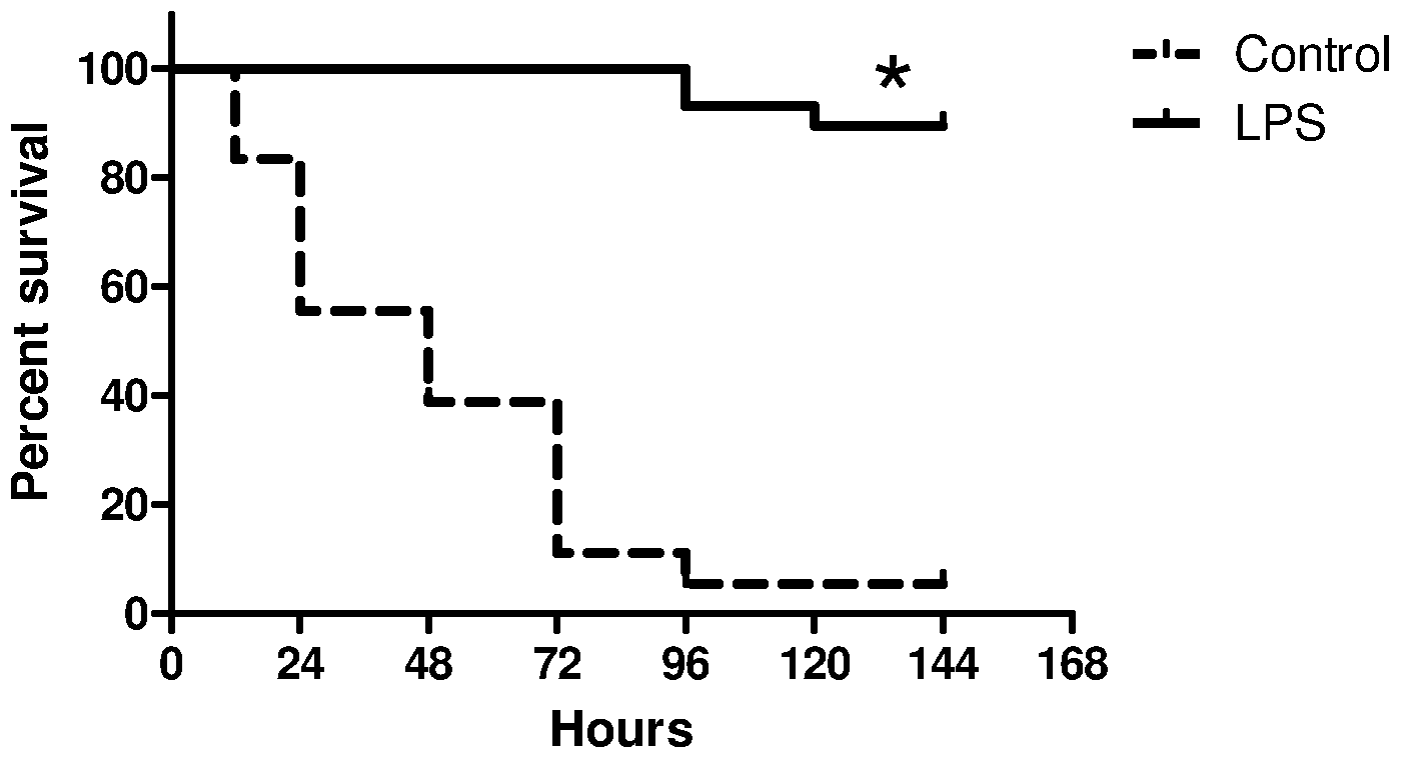
Survival curves for control (N = 18) and LPS pretreated mice (N = 29) following intravenous bacterial challenge with 2×10^6^ CFU of *S. pneumoniae* culture, *log-rank test, p<0.001.

Initial studies focused on whether survival from endotoxin tolerance could be extended to mice that received LPS pretreatment before challenge with *S. pneumoniae*. C57BL/6 mice were pretreated with PBS or LPS as described above and infected with *S. pneumoniae* 2 h after the last LPS injection. The onset of clinical symptoms such as piloerection and inactivity were observed earlier by 6–12 h in the control group, whereas the LPS pretreated group showed no symptoms of disease during the first 24 hours. Survival for LPS pretreated C57bl/6 was 90% (N = 26 of 29) compared to 0% (n = 0 of 18; p<0.001) in untreated controls (Figure 1). Median survival times for LPS pretreated and control mice were 96 and 48 hours respectively.

**Figure 2:**
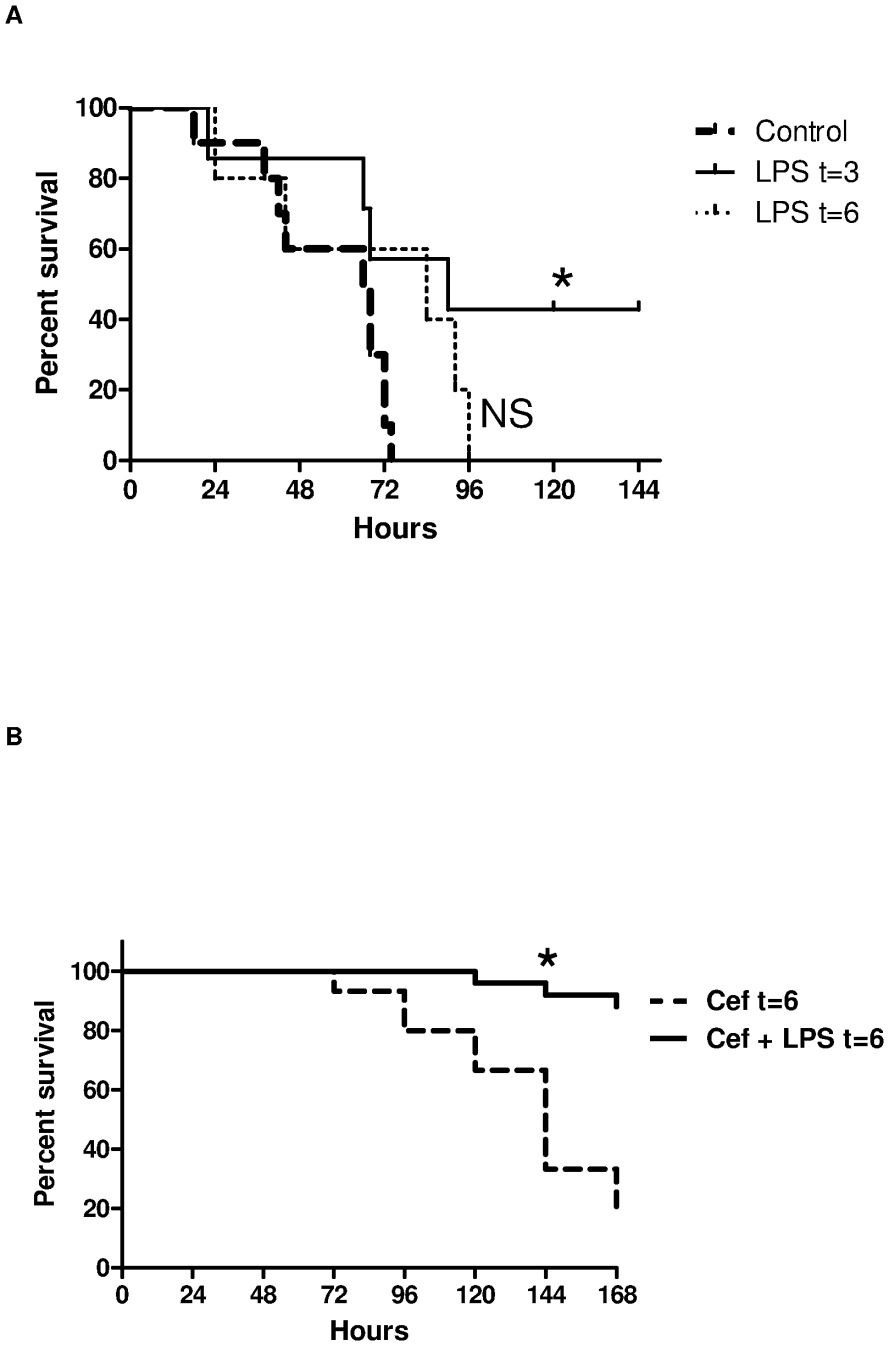
(A) Survival curves for C57BL/6 mice treated with LPS with or without ceftriaxone after bacterial challenge. Mice were treated with LPS 3(N = 7) or 6 hours (N = 5) and then every 12 hours after bacterial challenge for 2 days and compared with PBS treated controls (N = 10); (B) Survival curves for C57BL/6 mice treated with LPS and ceftriaxone (N = 20) 6 hours after bacterial challenge compared to controls (N = 18) that received ceftriaxone and PBS 6 hours after bacterial challenge, *log-rank test, p<0.001.

Having shown that LPS pretreatment improved survival in mice challenged with *S. pneumoniae* we set out to determine whether the same benefit would occur in mice that received posttreatment LPS. In the absence of antibiotic administration, LPS given 3 hours after *S. pneumoniae* infection improved survival to 42% (3 of 7) compared to 0% in controls (0 of 10; p = 0.03; Figure 2A). Administration of LPS alone at 6 hours or more after infection did not confer additional protection to infected mice. However, there was a greater survival benefit when LPS was administered 6 hours after *S. pneumoniae* infection with the addition of ceftriaxone administered 6 hours after pneumococcal challenge compared to mice that only received ceftriaxone 6 hours after infection (80%, 16 of 20 vs. 33%, 6 of 18; p<0.001; Figure 2B).

### Bacteriological analysis from blood and lungs

**Figure 3:**
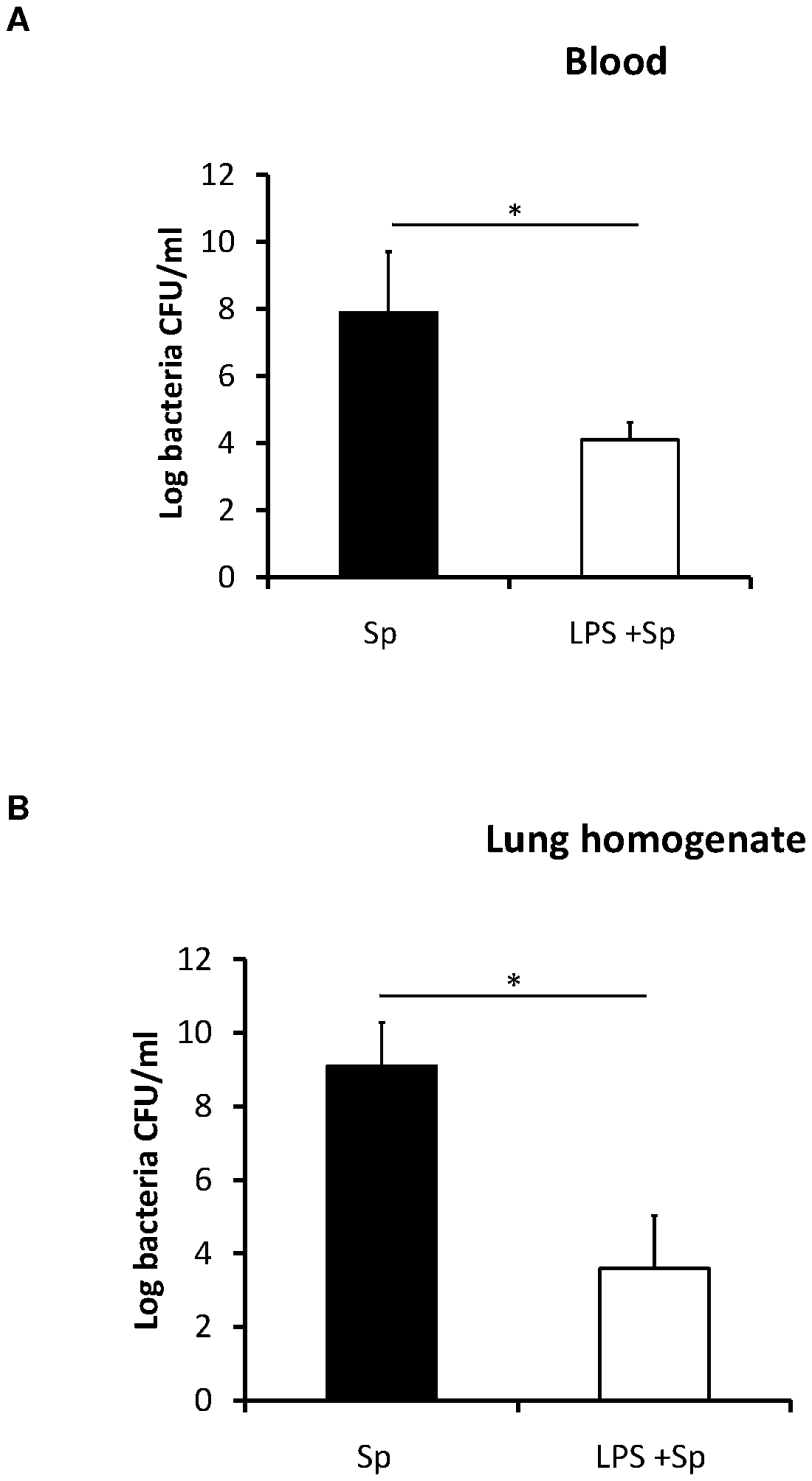
Bacterial clearance in (A) blood (N = 5–8 per group), and (B) lung homogenates (N = 3 per group) after *S. pneumoniae* challenge in LPS pretreated and PBS treated control mice. The results are expressed as the mean and standard deviations (SDs) are indicated by error bars, *t-test, p<0.05.

To determine whether the beneficial effects of LPS pretreatment were due in part to a change in infectious burden we measured the bacterial load in blood and lung homogenates of control and LPS pretreated mice after infection with *S. pneumoniae*. Systemic bacteremia (blood and lung) was reduced by at least four logs in LPS pretreated mice compared to the control untreated group (Figure 3A and 3B). A difference in bacterial load between LPS pretreated and control mice was observed as early as 6 hours after infection.

### The effect of pretreatment with low dose of LPS on blood chemistry in C57BL/6 mice

**Figure 4:**
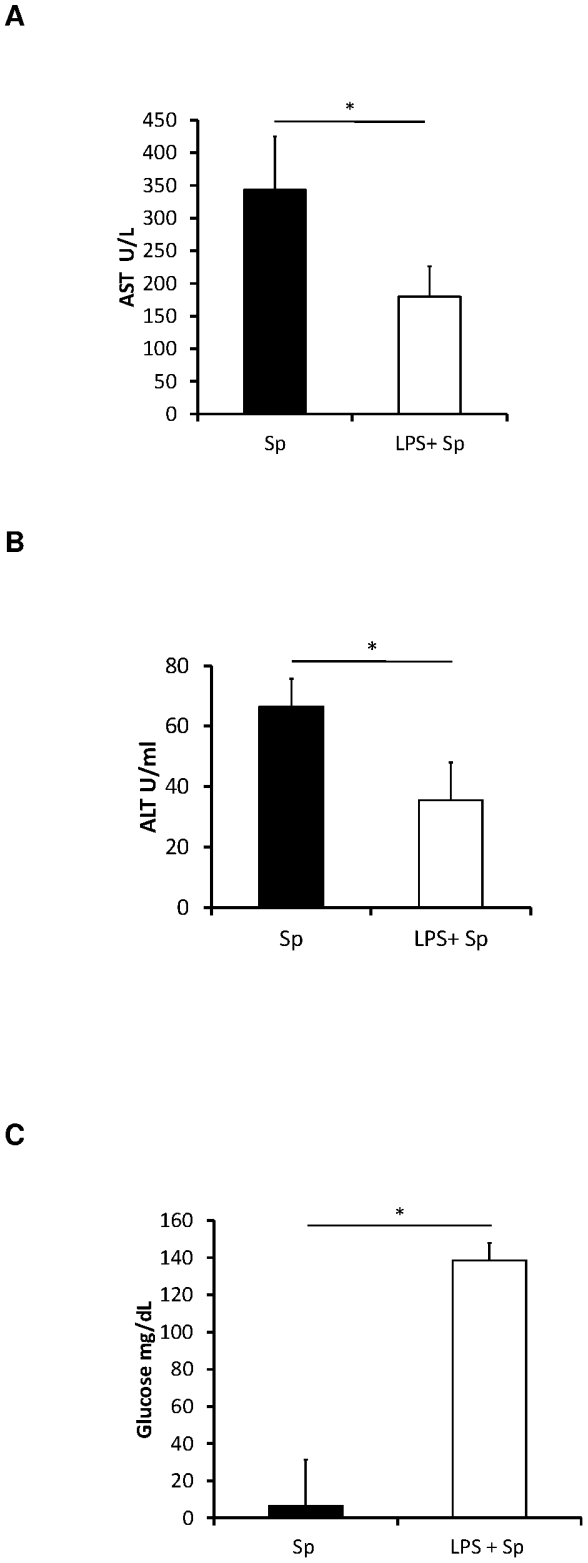
Mean ± SD serum (A) AST,(B) ALT, and (C) glucose concentrations at 6 hours after pneumococcal challenge in mice that received LPS pretreatment compared to controls (N = 4–6 per group), *t-test, p<0.05.

To determine the effect of LPS pretreatment on blood chemistry and determine if excessive liver injury may contribute to increased mortality, release of the hepatocyte-associated enzymes AST and ALT into the peripheral blood was examined. Serum activities of AST and ALT were statistically significantly reduced in the LPS pretreated mice compared with the control mice 6 hours after bacterial challenge (Figure 4A and 4B). LPS pretreatment reduced the AST and ALT to near baseline.

The blood glucose concentration was decreased at 6 hours after bacterial challenge in the control infected mice when compared with naïve mice. However, the blood glucose concentrations of the LPS pretreated mice at 6 hours after bacterial challenge were very close to those of naïve mice. The concentration of blood glucose in LPS pretreated mice was higher (near baseline) compared to the infected control mice 6 hours after bacterial challenge (Figure 4C). LPS pretreatment prevented hypoglycemia and increased survival.

### The effect of LPS pretreatment on cytokine production in C57Bl/6 mice

One of the hallmarks of endotoxin tolerance is the down-regulation of cytokine production therefore we determined whether this held true for mice challenged with *S. pneumoniae* after LPS pretreatment. Differences in cytokine and chemokine concentrations were noted as early as 3 hours after bacterial challenge which were more pronounced by 6 hours (Table 1).

**Table 1:** Mean serum concentrations of cytokines and chemokines at 3 and 6(n = 7–10 per group).

		T = 3 hours			T = 6 hours	
Cytokine or Chemokine	Sp alone	LPS	p-value	Sp alone	LPS	p-value
TNF-α	3301.3±2827.9	60±27.3	0.05	12254.8±5190	50.5±43.4	0.001
IL-1α	422.8±99.9	126.8±70.8	0.003	1148.1±21	87.8±87.7	0.001
IL-1β	64±114.9	48±15.6	0.05	798.3±483.8	3.8±7.7	0.001
IL-3	131.5±27.5	16.5±8.9	0.003	163.3±26.4	9.5±11.9	0.001
IL-4	72.5±40.6	31.5±19.2	0.50	106.8±58.4	2.8±6.2	0.002
IL-5	268.1±133.1	38.8±32.0	0.05	184.6±51.3	11.4±10.8	0.001
IL-6	9098±1094	236±92.1	0.001	9130±2812.8	87.5±117.4	0.001
IL-10	67.5±26.6	504±321.8	0.012	237.5±115.9	60±31.8	0.001
IL-12p40	11194±1429	1492±333.7	0.001	9908±4710.8	685.5±964.8	0.001
IL-12p70	1816.5±538.3	27.5±11.1	0.005	3161.5±2249.8	60±472.8	0.0001
IL-13	30.7±13.4	9.5±2.4	0.002	40±65.1	391±256.9	0.112
IL-17	407.5±145.5	24.5±13.1	0.05	353.8±228.6	100.8±252.2	0.14
Eotaxin	153.3±28.9	32±25.5	0.008	556±315	3.5±5.3	0.01
G-CSF	15333±355.7	8035±2776.2	0.03	10788±2054	4466±1726.1	0.03
GM-CSF	301±157.7	77.5±25.8	0.5	250.8±244.3	35±20.8	0.001
IFN-γ	1115±751.3	27±25.4	0.05	4168.5±3830	6.5±11.1	0.001
KC	16168±666.7	1076±289.7	0.001	16691±1523.6	452±156.8	0.001
MIP-1α	1751±1083.2	167.5±52.6	0.01	4227±1292.5	42±31.8	0.0001
MCP-1	9881±495.9	1412±726.3	0.01	10254±543.5	187±780.5	0.0001
RANTES	1266±312.3	200.8±65.6	0.0004	2841±1584.5	88.5±57.4	0.0009
MIP-1β	1320±571.9	97.3±0.7	0.009	2536±1275.5	18.8±23.6	0.02

### Requirements of TLR4 for activation of innate immune effectors by highly purified LPS

**Figure 5:**
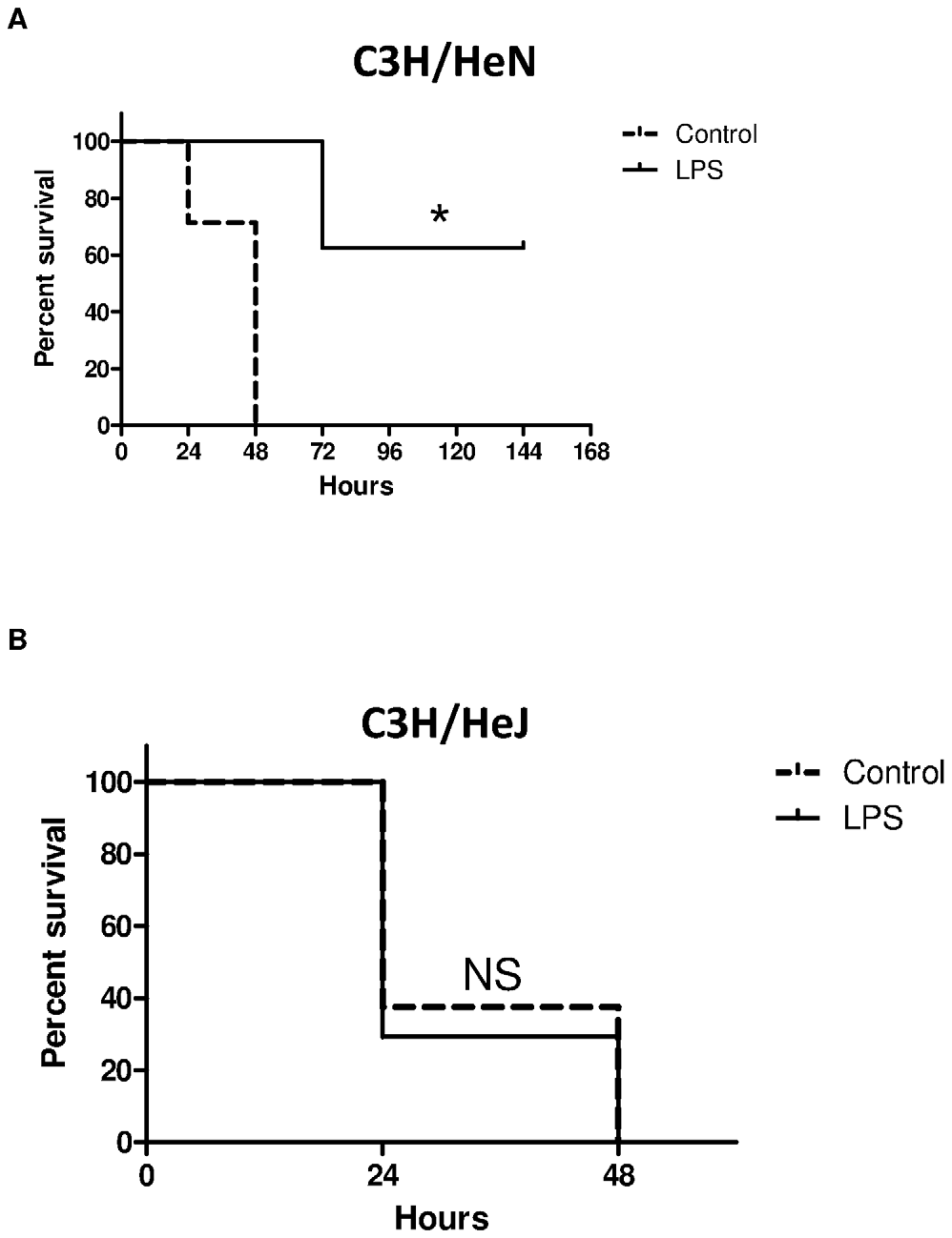
Survival curves for (A) C3H/HeN (N = 8) and (B) TLR4-deficient C3H/HeJ (N = 17) mice challenged with *S. pneumoniae* after pretreatment with LPS compared to PBS treated controls, *log-rank test, p<0.05.

**Figure 6:**
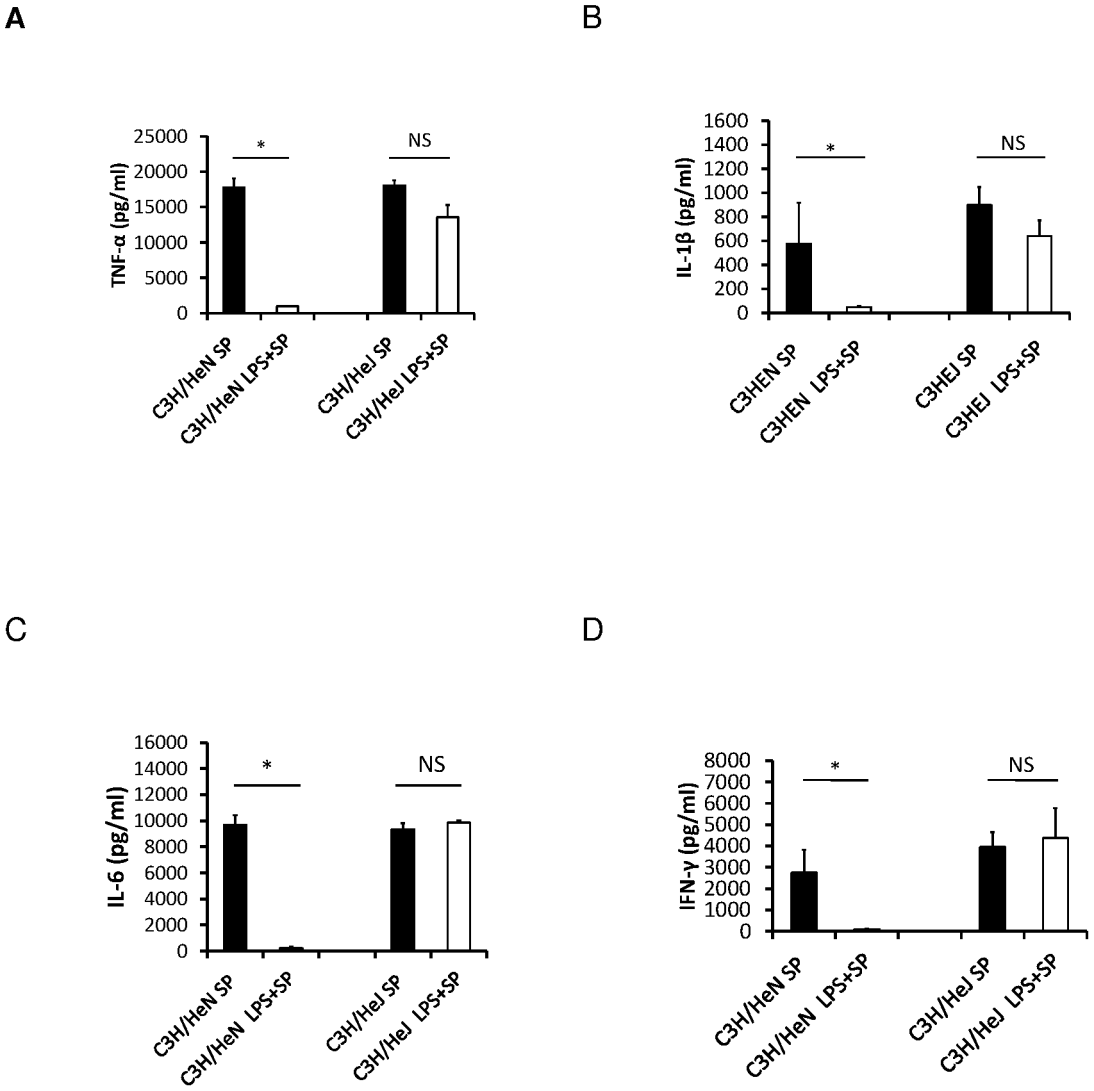
Serum concentrations of (A) TNF-α, (B) IL-1β, (C) IL-6, and (D) IFN-γ at 6 hours after bacterial challenge in LPS pretreated and PBS pretreated control C3H/HeN and C3H/HeJ mice (n = 5 per group), *t-test, p<0.05.

Having determined that LPS pretreatment could improve survival and decrease inflammation, transaminitis, and hypoglycemia, we focused on the mechanism of this phenomenon. LPS pretreatment improved survival in C3H/HeN mice (60%, N = 5 of 8, p = 0.009; Figure 5A) but not TLR4 deficient C3H/HeJ mice (0%, N = 0 of 17, p = 0.7; Figure 5B). Median survival times for control and LPS pretreatment C3H/HeN mice were 48 hours and 72 hours, respectively. In C3h/HeJ mice, the median survival times for LPS pretreatment and controls were similar at 17 and 24 hours respectively. Cytokine production was attenuated in C3H/HeN LPS pretreated mice compared to control mice. There was no statistical difference in the concentration levels of inflammatory cytokines between the LPS pretreatment and untreated C3H/HeJ mice (Figure 6A–D).

### The role of complement and T and B cells in LPS induced tolerance

**Figure 7:**
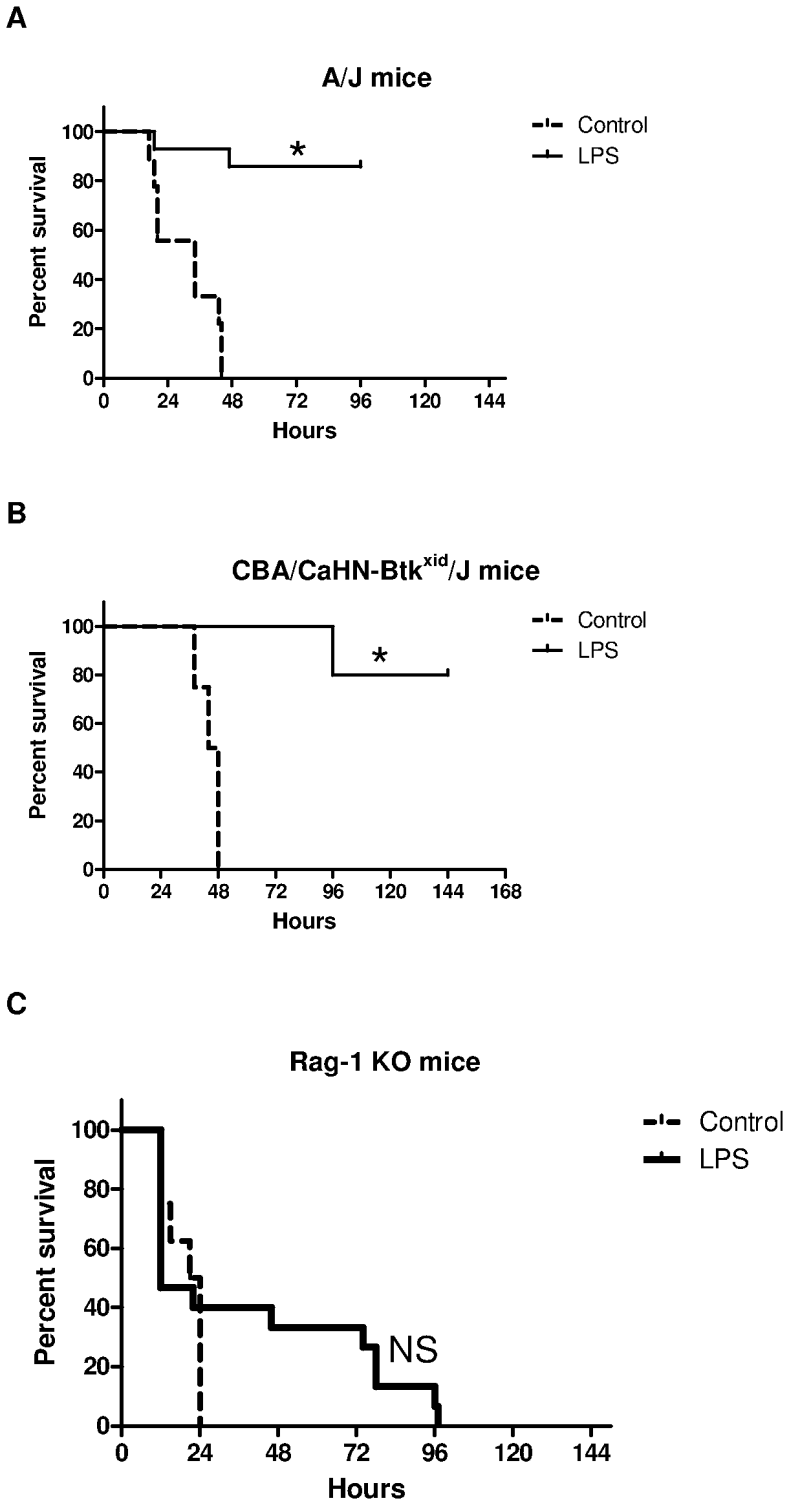
Survival curves for LPS pretreated and PBS control (A) A/J, (B) CBA/CaHN-Btk^xid/^/J, and (C) Rag-1 KO mice after challenge with *S. pneumoniae* (n = 4–14 mice per group), *log-rank test, p<0.001.

LPS pretreatment improved survival in both C5 deficient A/J mice (85%, N = 12 of 14, p<0.0001) and B cell dysfunctional CBA/CaHN-Btk^xid/^/J mice (80%, N = 8 of 10, p<0.005) compared to controls (Figure 7A and 7B). For A/J mice the median survival time for treated and untreated mice was 96 hours and 34 hours respectively and for CBA/CaHN-Btk^xid/^/J mice the median survival time was 140 hours and 46 hours respectively.

Since complement and B-cell function were not crucial to the survival benefit of endotoxin tolerance we also used Rag-1 knockout mice which are deficient in both T and B-cells to determine the role of T-cells. In contrast to mice with complement deficiency or B-cell dysfunction, LPS pretreatment did not improve survival in Rag-1 KO mice (0%, N = 0 of 15) when compared to the control group (0%, N = 0 of 8, p = 0.3; Figure 7C). These data support the pivotal role of T-cells in the survival benefit obtained from endotoxin tolerance during pneumococcal sepsis.

## Discussion

In this study we have shown the importance of T-cells in the protective effect of TLR4 stimulation in the setting of experimental sepsis. Furthermore, we have shown that TLR4 stimulation improves survival even when it occurs after the onset of pneumococcal sepsis. The first finding provides insight into the mechanism underpinning endotoxin tolerance and the second has implications for the clinical application of this intervention.

Endotoxin tolerance has previously been noted with LPS or live bacterial challenge after a preceding exposure to low doses of LPS. In our study we show for the first time that secondary exposure to LPS after primary pneumococcal infection still conveyed protection. This novel finding suggests that the mechanism of endotoxin tolerance is independent of the primary innate immune response that occurs with a gram positive pathogen such as *S. pneumoniae*. Another study showed that endotoxin tolerance improved bacterial clearance and improved survival in mice infected with *Staphylococcus aureus*, another gram positive pathogen [12]. It has also been shown that cell wall products from gram positive pathogens can improve survival from gram positive and gram negative pathogens [10], [11]. Our data along with others reveal a redundancy in the innate immune response to cell wall products which manifests as a reduction in inflammation whether or not TLR4 stimulation occurs before or after a live bacterial challenge. Whether tolerance also occurs with secondary TLR stimulation with peptidoglycan or another cell wall product after primary infection is not known.

An important finding in our study was that bacterial clearance occurred to a greater degree in the setting of endotoxin tolerance than in control mice. In TLR4 deficient mice, the bacterial burden was similar in mice that received LPS pretreatment compared to controls. The paradox of a reduction in bacterial burden in a hypo-inflammatory setting implies a control of bacterial infection beyond the hyper-inflammatory innate immune response that usually occurs. This control may occur through an increased neutrophil response as endotoxin tolerance leads to enhanced neutrophil extracellular trap formation and a more efficient bacterial clearance in mice [20]. There is also decreased neutrophil apoptosis in endotoxin tolerant mice which leads to improved bacterial clearance in septic mice [21]. An increase in Kupffer cell numbers and enhanced phagocytic activity of the liver in endotoxin tolerant mice also improves bacterial clearance in septic mice [22]. Since endotoxin tolerance led to increased survival in A/J and CBA/CaHN-Btk^xid/^/J mice deficient in C5 complement and B cell function respectively, the increased bacterial clearance is likely complement and antibody independent.

It has recently been shown that in the early phase of human severe sepsis there is an increase in the percentage of activated (CD69+) T-cell subsets compared to healthy controls [23]. Also, in patients with severe sepsis increased granzyme levels in cytotoxic T-cells are associated with disease severity. Additionally, T-cells are activated and recruited to the lungs of mice early after infection with *S. pneumonia*e [24]. Furthermore, MHC class II-deficient mice which are largely devoid of CD4+ T-cells and CD4+ T-cell antibody depleted or cyclosporine treated mice exhibited a significantly higher median survival time than wild-type control mice [25]. This survival benefit was accompanied by a decrease in serum inflammatory cytokines. In another study, reduction of αβ but not γδ T-cells improved survival after cecal ligation and puncture [26]. Taken together, these data suggest that T-cells play an important role in the mediation of inflammation, morbidity, and mortality from sepsis. Our data reveal a crucial role for T-cells in the mechanism of endotoxin tolerance to sepsis. Although the mechanism of endotoxin tolerance is not fully delineated, in a relevant model of asthma, endotoxin tolerance attenuated airway allergic inflammation by suppression of the T-cell stimulatory effect of dendritic cells [27].

In our experiments the benefit of endotoxin tolerance in survival from pneumococcal sepsis was dependent on both T cells and TLR4. TLRs are important mediators of inflammation and are increasingly associated with T-cell biology [28]. Both CD4+ T-cells and CD8+ T-cells express appreciable levels of mRNA for various TLRs including TLR4 [29], [30]. Regulatory T-cells (Tregs) express TLRs which are activated by endotoxin and TLR4 hyper function *in vivo* potentiates Treg function to curtail TLR4-dependent autoimmunity [31], [32]. Endotoxin tolerance can induce a marked resistance of lymphocytes against cell death during the subsequent period of sepsis [33]. In addition, endotoxin tolerance produces an increased differentiation of T-lymphocytes to TH1 and TH2, with a TH1 differentiation predominance [33]. Therefore, improved survival after pneumococcal challenge in the setting of endotoxin tolerance may be attributed to alterations in T-cell function which were manifested by differences in expression of cytokines and chemokines in blood. Further work is required to determine which T-cell subsets and signaling pathways are required to potentiate the effects of endotoxin tolerance.

## Conclusions

This study provides evidence that induction of endotoxin tolerance, despite reducing cytokine production, improves host defense against infection with a virulent strain of *S. pneumoniae*. Furthermore, endotoxin tolerance can occur after the onset of pneumococcal sepsis. This finding has obvious clinical importance for episodes of acute sepsis. TLR4 agonist activity can therefore be potentially exploited to provide short-term resistance to infectious challenge. However, caution exists regarding the efficacy of endotoxin tolerance in the hypo-inflammatory late stages of sepsis which is thought to be permissive to opportunistic and second-hit infections [34]–[37]. Nonetheless, our data provide novel insights into the application of endotoxin tolerance as a therapeutic intervention for pneumococcal sepsis as well as the role of T-cells in this phenomenon. Further work is required to delineate the best use of this immune modulation for the best clinical effect as well to better understand the role of T-cells in endotoxin tolerance.
